# Enhancing metabolomic data analysis with Progressive Consensus Alignment of NMR Spectra (PCANS)

**DOI:** 10.1186/1471-2105-11-123

**Published:** 2010-03-09

**Authors:** Jennifer M Staab, Thomas M O'Connell, Shawn M Gomez

**Affiliations:** 1 Department of Computer Science, University of North Carolina at Chapel Hill, Chapel Hill, North Carolina, USA; 2 Curriculum in Bioinformatics and Computational Biology, University of North Carolina at Chapel Hill, Chapel Hill, North Carolina, USA; 3 Division of Pharmacotherapy and Experimental Therapeutics, School of Pharmacy, University of North Carolina at Chapel Hill, Chapel Hill, North Carolina, USA; 4 Hamner-UNC Center for Drug Safety Sciences, Research Triangle Park, North Carolina, USA; 5 Department of Pharmacology, University of North Carolina School of Medicine, Chapel Hill, USA; 6 Joint Department of Biomedical Engineering, University of North Carolina School of Medicine, Chapel Hill, North Carolina, USA

## Abstract

**Background:**

Nuclear magnetic resonance spectroscopy is one of the primary tools in metabolomics analyses, where it is used to track and quantify changes in metabolite concentrations or profiles in response to perturbation through disease, toxicants or drugs. The spectra generated through such analyses are typically confounded by noise of various types, obscuring the signals and hindering downstream statistical analysis. Such issues are becoming increasingly significant as greater numbers of large-scale systems or longitudinal studies are being performed, in which many spectra from different conditions need to be compared simultaneously.

**Results:**

We describe a novel approach, termed Progressive Consensus Alignment of Nmr Spectra (PCANS), for the alignment of NMR spectra. Through the progressive integration of many pairwise comparisons, this approach generates a single consensus spectrum as an output that is then used to adjust the chemical shift positions of the peaks from the original input spectra to their final aligned positions. We characterize the performance of PCANS by aligning simulated NMR spectra, which have been provided with user-defined amounts of chemical shift variation as well as inter-group differences as would be observed in control-treatment applications. Moreover, we demonstrate how our method provides better performance than either template-based alignment or binning. Finally, we further evaluate this approach in the alignment of real mouse urine spectra and demonstrate its ability to improve downstream PCA and PLS analyses.

**Conclusions:**

By avoiding the use of a template or reference spectrum, PCANS allows for the creation of a consensus spectrum that enhances the signals within the spectra while maintaining sample-specific features. This approach is of greatest benefit when complex samples are being analyzed and where it is expected that there will be spectral features unique and/or strongly different between subgroups within the samples. Furthermore, this approach can be potentially applied to the alignment of any data having spectra-like properties.

## Background

Continuing technological advances are providing rich data sets quantifying an increasingly broad range of biological processes. Obvious examples include the use of microarrays for the quantification of mRNA levels and mass spectroscopy for the identification of protein states and their interactions. Coinciding with these technological developments are computational approaches for the extraction, organization and analysis of these data. The application of improved experimental methods in combination with tailored computational approaches is providing a major driving force in the development of a more global, systems perspective of biological function and disease.

Metabolomics, also referred to as metabonomics, similarly provides a comprehensive picture of biological function by focusing on quantitative measurement of metabolites in biological fluids, cells or tissues [1,2]. The two major analytical platforms used in metabolomics are nuclear magnetic resonance (NMR) spectroscopy and mass spectrometry (MS), the latter typically being preceded by either liquid or gas chromatography (LC/MS and GC/MS respectively). The ultimate goal of these methods is to extract accurate and quantitative information as to the identity, state and amount of detected metabolites. Increasingly common in metabolomic studies is the analysis of a large number of samples, where the resulting data is analyzed using multivariate methods such as principal components analysis (PCA). Such analyses typically require significant preprocessing of the data. In particular, it is imperative that signals for a given compound appear at the same location in all spectra. Signal locations can vary significantly, however, as in the case of LC/MS where small deviations in the chromatographic retention time can arise from variation in instrumental parameters such as flow rate, gradient slope and temperature. In NMR spectra, the peak location can vary due to differences in pH, ion content and the concentration of metabolites. For both of these methods, this variability has to be overcome in order to provide a consistent set of spectra for analysis.

The most common method of addressing variability across spectra is through binning, a procedure that involves dividing the spectra into small windows and taking the area under the curve for each window as the final intensity [3,4]. Ideally, these windows will be large enough to encompass the peak drift, but not so large as to include many peaks in a single bin. The latter consequence is unavoidable in crowded spectra and thus there is the potential for significant loss of information when binning, for example by including peaks belonging to multiple compounds within a single bin. Alternatives to binning typically involve some form of peak alignment procedure. For LC/MS methods, a number of algorithms have been developed to align similar peaks across a set of chromatograms (e.g. [5] and recently reviewed in [6]). Similarly, several algorithms have also been recently developed to align peaks in sets of NMR spectra [7-11]. In this paper we describe a novel peak alignment method for NMR that is specifically tailored to the demands of large and disparate metabolomics datasets.

Current advanced NMR alignment methods such as fuzzy warping [7], Bayesian alignment [8], Recursive Segment-Wise Peak Alignment [10], peak alignment by FFT [5,11] and peak alignment using reduced set mapping without recursive target update [9], are based on the use of a template spectrum to help align a set of spectra. Choosing a template typically involves either selecting a single sample spectrum that appears most like the others as determined by some measure of similarity, creating an "average" spectrum, or by choosing a reference spectrum not contained within the sample. All remaining sample spectra are then aligned to this selected template using some form of pairwise alignment algorithm. A significant problem with the template approach is that there can be a great amount of variability between any two spectra. Part of this difference arises due to the previously described chemical shift variation. In addition, significant differences arise due to the existence of disparate groups within the data; for instance, inter-group variation between control and treated groups, subpopulation differences within these groups, etc. There may often be a priori knowledge of general subgroups, but one of the goals of metabolomics is to discover new subgroups such as different types of responders in drug or toxicity studies; by definition, templates for such groups are not known beforehand. Thus in such cases, the use of a template can significantly complicate downstream analyses.

Here we describe a novel approach for the alignment of NMR spectra that is based on the creation of a consensus spectrum alignment through integration of pairwise spectrum comparisons and referred to as PCANS hereafter (Progressive Consensus Alignment of Nmr Spectra). To our knowledge, this is the first such consensus approach applied to the alignment of NMR spectra. This approach has several advantages that include the ability to align spectra with significant amounts of noise in chemical shift position, peak height and peak width. By using peaks as the basis for alignment we maintain the maximally informative set of information existing within a set of spectra. As a result, the existence of subgroups within a set of spectra can be identified since group-specific peaks are maintained in the final alignment.

We characterize the performance of this approach by aligning simulated NMR spectra which have been provided with user-defined amounts of chemical shift variation as well as inter-group differences as would be observed in control-treatment applications. Moreover, we demonstrate how our method provides better performance than either a template-based alignment or binning. Finally, we further evaluate this approach in the alignment of real mouse urine spectra and demonstrate its ability to improve downstream statistical analyses such as PCA and OPLS models commonly used in metabolomics analyses.

## Results and Discussion

**Figure 1:**
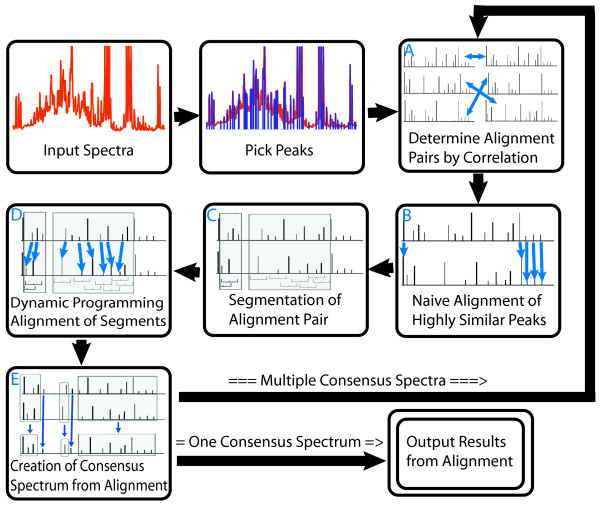
**Overview of the PCANS Alignment Process**. The alignment process loops through multiple iterations of pairwise alignment until achieving a single consensus profile. See text for further details.

While the alignment method we propose consists of several steps which are described in detail in Methods, we provide a brief overview here. As outlined in Figure 1, our approach begins by first characterizing each individual spectrum by defining its peaks. The process of picking peaks can be done through a variety of methods and we have used a straightforward approach that uses the derivative of the spectrum, and other associated properties for discerning peaks. The resulting set of peaks contains the location, height and width of all peaks in a spectrum, referred to as the peak profile. These features comprise the main information content that is used in the interpretation of NMR spectra. This approach allows each NMR spectrum to be represented by a much smaller collection of data points than if we used the full resolution of the acquired spectrum. For example, our experimental urine spectra were collected with 16384 points, but the peak picking process found that the spectrum contained less than 500 significant peaks. It must be noted that peak picking may result in loss of information if some peaks are not picked. The peak picking algorithm is under active development to insure that peak information is not lost due to features such as low signal to noise or spectral crowding. Future work will also consider spectral features such as multiplet structure to provide more accurate peak profiles.

**Figure 2:**
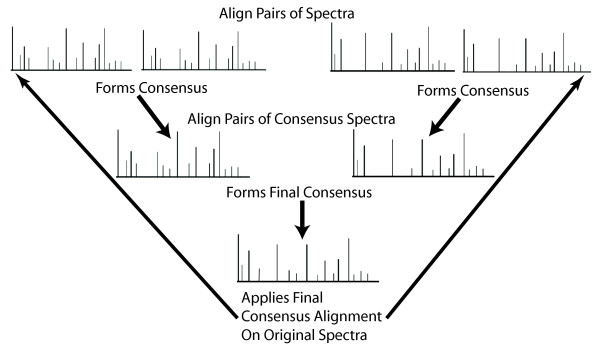
**Final Consensus Profile Formation**. Pairwise alignments are progressively combined together through the alignment of consensus profiles to form a final consensus profile. This profile is then used to adjust the chemical shift positions of the peaks from the original input peak profiles to their final aligned positions.

In the next step of the process, pairs of peak profiles are chosen for alignment, where the most similar profiles are determined through pair-wise statistical correlation (Figure 1A). Thus we start by aligning the most similar pairs of profiles to each other first. Each of these pairs of profiles is then aligned through a series of progressively more rigorous steps that begins with the naive alignment of the most highly similar peaks (Figure 1B-D). This naive alignment establishes aligned regions of high identity separated by segments that cannot be so readily aligned. These segments, bordered on either side by high-confidence aligned regions, are then aligned through a dynamic programming algorithm where the alignment score is based on chemical shift position, peak height and peak width. Note that only the peak location is altered throughout the alignment process and that peak height and width remain unaltered. Following this first pairwise alignment, a single consensus profile is created (Figure 1E). This process is then repeated, first for each set of pairs and then progressively for all of the generated consensus profiles. At the end of this process a single representative consensus profile is generated which defines the final alignment (see Figure 2). The final output consists of the set of input profiles with their respective peaks aligned to this final consensus alignment.

### Alignment of simulated spectra

As "gold-standard" completely characterized NMR spectra for use in validation are not available, we used a simulation approach for generating peak profiles that could then be used to assess the performance of the alignment methods. In particular, the use of simulated profiles allows us to determine whether or not two or more peaks aligned through our algorithm should actually be aligned with each other, and if not, which other peaks they should be aligned to. It also allows us to introduce defined amounts of noise, either in the form of chemical shift variation, peak height, peak width, or randomly introduced "noise" peaks into each profile and measure their effect on alignment accuracy. As we wished to generate NMR profiles that were as realistic as possible, our simulated profiles were composed of a subset of peaks picked from an actual mouse urine spectrum (see Methods).

As a test of our alignment approach, we attempted to align simulated profiles under a variety of noise conditions. In these tests we generated two sets of profiles consisting of 32 profiles each, where each set was based on a different template. Each template consisted of a total of 50 peaks, 13 of which were unique to each group, allowing us to look at the effectiveness of alignment in the presence of inter-group variation. In addition to the differences derived from the peaks specific to each group, predefined amounts of chemical shift, peak height and peak width variation were also introduced before alignment. Finally, 50% of the profiles in each group had from 1 to 4 additional noise peaks inserted at random positions within each profile.

**Figure 3:**
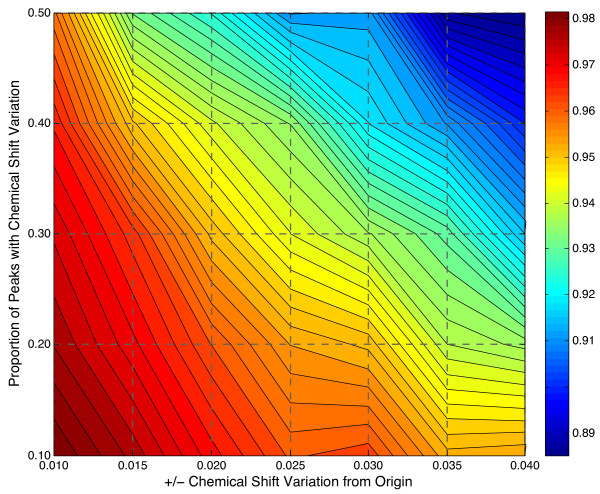
**Accuracy of Alignment with Simulated Peak Profiles**. The x-axis indicates ± range of chemical shift variation and the y-axis indicates proportion of peaks per profile that experienced chemical shift variation. The graph depicts accuracy as indicated by the colorbar on the right, where PCANS achieved accuracies between 98.4% and 88.5%. Besides chemical shift position, both relative intensity and width were randomly perturbed by ± 10% of the origin for 25% of the peaks within each profile and 50% of the profiles had 1-4 noise peaks randomly added.

The effects of chemical shift variation on alignment accuracy are shown in Figure 3 where, in addition to chemical shift variation, 25% of peaks were subject to noise of ± 10% in peak height and/or peak width at half-height. The contribution of chemical shift, peak height and width to the alignment score were kept equal in this and all other tests as this combination was found to be highly robust. Sensitivity to the choice of these weighting parameters is shown in Additional File 1: Figure S2. In Figure 3 we see that the accuracy of alignment is highly robust to chemical shift variation as can be seen by the slow decrease in accuracy with increasing variation. Here, alignment accuracy is calculated by dividing the number of peaks correctly aligned by the total number of peaks. Alignment is similarly robust to increases in the proportion of peaks subjected to such variation. In fact, a nearly 90% accuracy is maintained despite 50% of peaks experiencing variation of up to ± 0.04 ppm. The maximum standard deviation is ± 0.033 and the corresponding map of deviations is shown in Additional File 1: Figure S1. While we used a window of ± 0.04 ppm in the alignment of individual peaks, this is a user-defined quantity that can be changed to suit the underlying data.

We also compared the accuracy of our alignment method between our consensus approach and the use of a template. Again, we started with two sets of profiles, with each set consisting of 32 profiles and 50 peaks, with 13 peaks unique to the set. Variation in chemical shift position (± 0.02 ppm) was introduced for 50% of the peaks. Peak height and width noise (25% of peaks affected with ± 10% variation) was also independently introduced. As before, 50% of the profiles in each group had 1 to 4 noise peaks inserted at random chemical shift positions.

We iteratively chose one of the sixty-four peak profiles as the template to which all the other profiles were aligned. Thus this approach differs from the PCANS alignment method only in the fact that it uses a representative profile as a template for use in aligning the other peak profiles; all other steps are identical including the dynamic programming alignment of peaks. Over all 64 possible templates, the average accuracy using this approach was 84.4% with 99% confidence intervals of 84.02% and 84.68%. The best single template had an accuracy of 87.5%. In contrast, PCANS had a 93.9% accuracy (PCANS generates only one answer so there are no error bars in this case).

**Figure 4:**
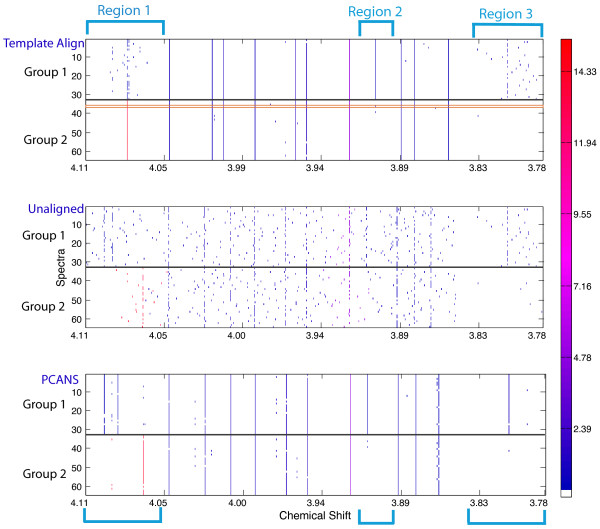
**A Sample Region of Simulated Peak Profiles Before and After Alignment**. Alignment is shown with either PCANS-aligned or template-aligned peak profiles. Short, individually colored bars indicate a profile's peaks. Peak profiles were simulated from two groups having group-specific peaks, with 32 profiles in each group. The colorbar on the right indicates relative height (intensity) of the simulated peak profiles. The horizontal orange rectangle in panel A indicates the best overall individual peak profile that was used as the template for this alignment. Regions indicated by vertical cyan rectangles depict cases where Group 1 has unique peaks that differentiate it from Group 2.

A representative region of an alignment is shown in Figure 4 where the template generating the highest accuracy (87.5%) was used to generate the shown template-based alignment. Differences between the template, unaligned and PCANS alignments can be readily observed. For example, three regions are highlighted that have peaks unique to Group 1. In Region 1 of the unaligned spectra (center row), it is possible to pick out by eye the existence of two likely peaks in Group 1 with no nearby corresponding peaks in Group 2. In addition, a peak unique to Group 2 is also visible in this region. In the template alignment (top row) the two peaks of Group 1 could not be aligned, as the best overall template did not contain associated peaks in these locations. In addition, the template did have a peak in Group 2, but at the wrong location, forcing alignment of the unique Group 2 peak to a shifted location. In contrast, PCANS correctly aligned the Group 2 peak (bottom row). Furthermore, the two peaks unique to Group 1 were also successfully aligned. Note that the rightmost peak of the pair appears to be shifted to the right. This is due to the variation present within the unaligned set of peaks. Aligning noisy spectra containing peaks with varying chemical shift position with PCANS results in the alignment of peaks at their median chemical shift position. This provides a robust estimate of peak position despite potentially significant amounts of spectral noise.

In Region 2 of the template alignment, we see a well-aligned peak for Group 1. However, as we are using simulated data, we know that the position of this alignment is centered at a nearby noise peak within the template profile and inspection of the unaligned profiles also shows no obvious peak. The correct result is shown in Region 2 of the PCANS alignment. This incorrect alignment occurs because the "best" template happens to contain a nearby noise peak that is used as the basis for alignment of all other profiles.

Finally, in Region 3 (unaligned) we see strong indications of a peak in Group 1 as well as alignment of this peak with PCANS. However, in the template alignment we see no obvious change relative to the unaligned profiles. This is due to the fact that the template profile had no peaks in this region and thus none of the identified peaks could be aligned. The fact that they are present at all in the final alignment is due to the PCANS-portion of the algorithm (non-template), which allows these orphan peaks to pass through to the final alignment regardless of whether or not they are found in the template. Overall, this example demonstrates the inherent pitfalls and challenges that arise with any alignment method that is based on the concept of a template or standard spectrum.

### PCA analysis of simulated spectra

**Figure 5:**
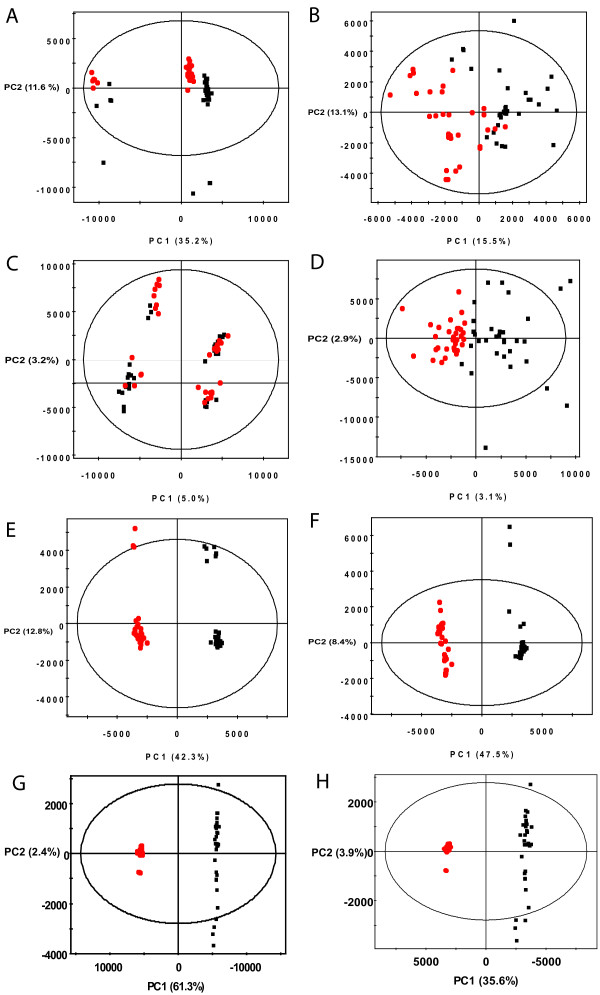
**PCA Analysis of Simulated Peak Profiles**. Displays binned (A & B), unaligned (C & D), PCANS aligned (E & F), and template aligned (G & H) simulated peak profiles, with (B, D, F & H) and without (A, C, E & G) outliers removed.

To further demonstrate how spectral alignment with PCANS can improve downstream data analysis, we performed a PCA analysis of unaligned, binned, PCANS-aligned and template-aligned peak profiles (see Figure 5). This data set consists of 1368 unique peaks when unaligned, 216 unique peaks after template alignment, 91 unique peaks after alignment with PCANS and 46 chemical shift position bins. PCA analysis of a perfect alignment of the data would show two tightly clustered groups with the only variance due to the small number of noise peaks added to each of the groups. The scores plot resulting from a standard binning procedure with uniform 0.04 ppm bin widths is shown in Figure 5A. This plot displays three distinct groups along with several outliers on the bottom half of the plot. The simulated peak profiles contain two large peaks that were modeled after the creatinine peaks that are found in urine. Thus the separation of the three clusters, as well as the outliers in this model, are largely due to inclusion of the creatinine peaks into four separate bins. The corresponding loadings plot for this scores plot is given in the supplemental information (Additional File 1: Figure S3). Figure 5B shows the scores plot that results from excluding those four bins. The separation between the groups is clearer, but the clustering is still rather diffuse.

Similarly, Figure 5C shows the PCA scores plot of the unaligned peak profiles. In this case the four clusters do not distinguish the groups, and are again based upon differences in the peak positions of two large creatinine peaks. As with the binning example, removal of these two peaks leads to a clearer discrimination of the groups, but again with a diffuse clustering as observed in Figure 5D.

In contrast, Figure 5E shows the results of PCA analysis after alignment with PCANS. The separation between the groups along the first principal component is complete and the clustering is very tight. The outliers from each group near the top of the plot are again due to one creatinine peak that did not get aligned with the rest. Removal of this peak from the analysis lead to the scores plot in Figure 5F. Although there are still a small number of outliers, the separation between the groups along the 1st principal component is excellent and this PC explains nearly fifty percent of the variance in the data.

The final comparison is with the template aligned peak profiles. Figure 5G displays a very good discrimination of the groups, with the control groups being very tightly clustered. This is reasonable as the template was chosen from that group. If, as with the other plots, the creatine and creatinine peaks are removed, the separation looks quite similar but the percent variation explained by the first principal component decreases by nearly half. Compared with the PCANS plot, the control group is more tightly clustered, but the treated group is less well so. Furthermore, the first principal component of the PCANS alignment explains 46.5% as opposed to 35.6% of the variation. In this rather simple example of only two groups, the PCANS alignment does have some advantages, but the benefits would be expected to be much greater in a more complex situation which has more than two groups.

### Alignment of Mouse Urine Spectra

**Figure 6:**
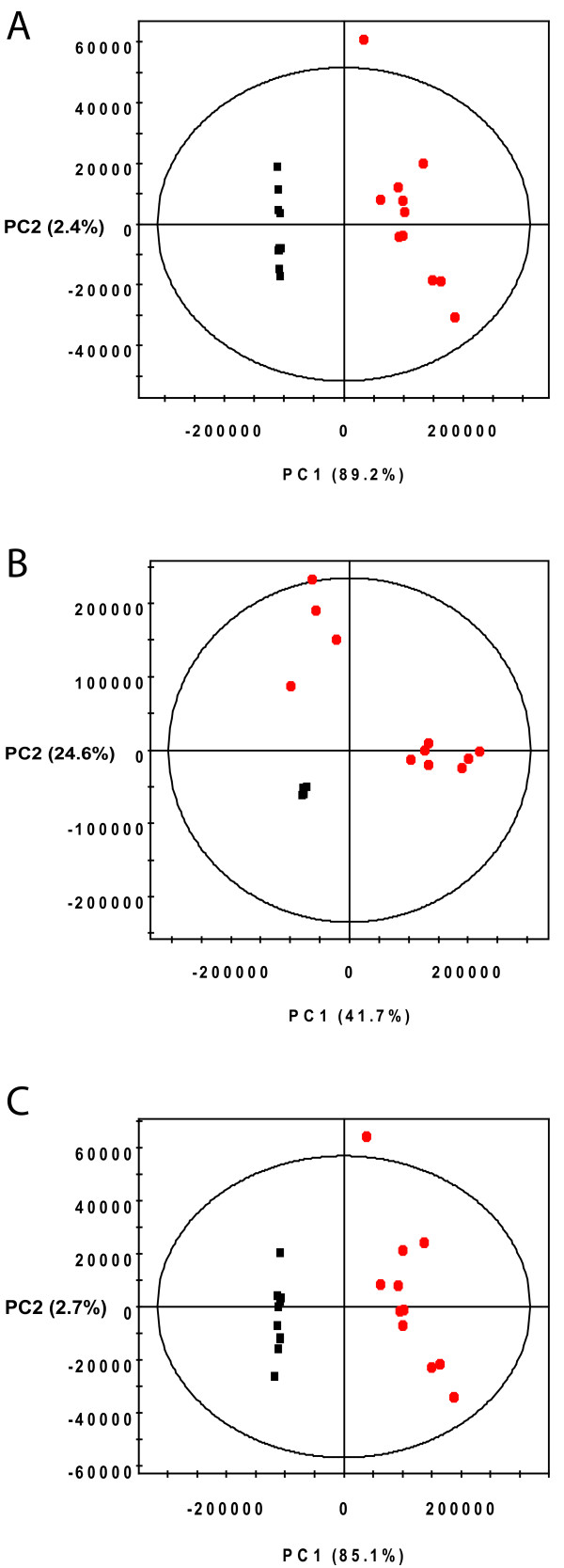
**PCA Analysis of Mouse Urine Spectra**. Displays binned (A), unaligned (B), and aligned (C) mouse urine peak profiles.

To demonstrate the utility of PCANS on real data, we applied our approach to the alignment of twenty-two mouse urine spectra from a recent study of ethanol toxicity [12]. In this study, half of the samples were taken from mice receiving chronic ethanol treatment and the other half were from controls, with results from PCA analysis shown in Figure 6. In Figure 6A, the data was analyzed using standard binning with 0.04 ppm bins, resulting in 152 bins across the spectrum. As can be seen, the correct separation of the data into two groups is discovered, largely due to the presence of ethanol and ethylglucuronide in the spectra of dosed mice (see Additional File 1: Figure S4). This positive result indicates that the chemical shift drift amongst these peaks is generally smaller than the applied bin width (i.e. the bin-widths are appropriately set to capture the chemical shift variation within these samples). Figure 6B shows the data prior to alignment. In its unaligned form, this dataset consists of 1496 unique chemical shifts. In this case the control samples are very tightly clustered (black points) and the major variation in the data appears in the dosed spectra. Again, the corresponding loadings plot shows that the separation of the dosed group into two clusters is predominantly due to the small differences in the ethanol and ethylglucuronide peaks (Additional File 1: Figure S4B). After peak alignment with PCANS, the number of unique chemical shifts is reduced to 483. The scores plot looks remarkably similar to that generated from the binned data and the percent variances for both of the PCs in these models is very similar (see Figure 6C).

Given the similarity between the binned and aligned data, the advantage of alignment with PCANS may not be obvious. However, it should be emphasized that the information content of the PCANS-aligned data is over three-fold larger than that of the binned data. More specifically, the intensity of 483 individual peaks is represented in the PCANS-aligned data, while the binned data encodes only 152 variables, many of which are influenced by (i.e. containing) multiple peaks. We can better observe the advantage that PCANS alignment provides through this added information by looking at the results of a supervised OPLS analysis.

**Figure 7:**
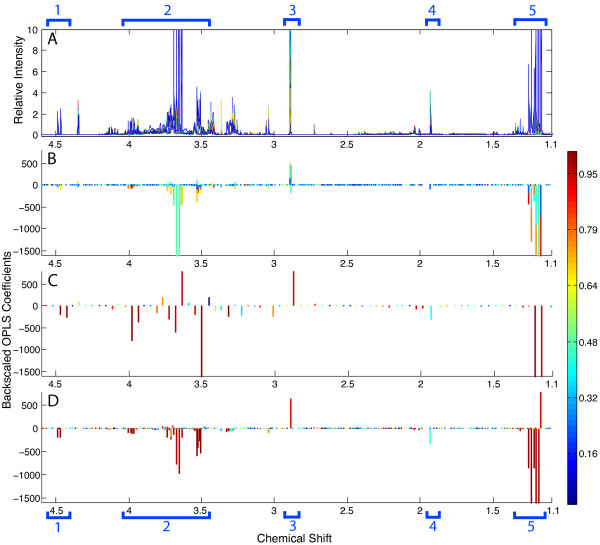
**OPLS Analysis of Mouse Urine Spectra**. Displays unaligned (B), binned (C), and aligned (D) OPLS coefficients from mouse urine peak profiles. Panel A depicts the unaligned peak profiles that correspond to the OPLS analysis. Peaks associated with glucose are located in bracket 1, ethanol and ethyl glucuronide in brackets 2 & 5, TMA in bracket 3, and acetate in bracket 4.

Figure 7 shows the OPLS loadings coefficients plots using data from each of the three data sets, with original spectra from dosed and control samples superimposed in Figure 7A[13,14]. Figures 7B-D show the back-scaled loadings coefficients such that the spectral features that are higher in the control group are positive and those that are higher in the ethanol treated group are negative. The color relates to the strength of the correlation, with red being the strongest. In Figure 7B, we see the OPLS coefficients prior to alignment and observe very weak correlation between peaks (blue-green colors in the Figure). In addition, several spectral features are largely missed (Regions 1 and 4 indicated in brackets at the bottom of the figure) or only weakly identified (Regions 2, 3 and 5). Application of OPLS analysis to binned data is shown in Figure 7C. The decrease in the data density due to peak consolidation into bins can be observed in this figure by the sparseness of data along the x-axis. While some of the correlations are higher, there are inappropriate assignments between groups as can be observed in the positively-valued peaks in Region 2. While the binned data shows the importance of the ethanol peaks in distinguishing the groups, interestingly the signals from ethylglucuronide are very weak.

In contrast, Figure 7D shows the loadings coefficients after PCANS alignment. As can be seen along the x-axis, data density (and thus information content), is significantly higher than with binned data. In addition, the signals from ethanol and ethylglucuronide are all very strong with colors indicating a very high confidence. Also note that while template alignment would perhaps perform similarly with regard to generating strong correlations for aligned peaks, it suffers from the earlier described difficulties that will lead to loss of peaks defining group/inter-group differences, loss of unique peaks, and alignment of different, but close peaks to nearby peaks within the template. These results demonstrate the value alignment with PCANS and its ability to enhance the information content relative to the standard binning protocol.

## Conclusions

The increasing scale and complexity of metabolomics studies is driving the need for improved computational tools for data integration and analysis. We have described our PCANS approach which was developed to address the need for multiple spectrum alignment where noise in the form of chemical shift variation and deviations in peak properties is present, along with significant sample complexity.

In metabolomic analyses there are often multiple groups of spectra, such as control and treated groups, which may not be appropriately aligned with any algorithm that is primarily based upon the use of a template. For instance, the peaks from the exogenous metabolites that are present in the treated group may not be well aligned using a template from the control group. Similarly, alignment of the control spectra could be confounded by the presence of the exogenous peaks in the template. Even when a specific group (e.g. the treatment group) can be reasonably well aligned by a representative template spectrum, metabolomics is also being increasingly used to determine multiple responder phenotypes wherein the treated group may contain several subgroups characterized by distinctly different spectra. Thus a significant advantage of PCANS over the use of template-derived methods is that it is a fully unsupervised method and can be used to align spectral data containing multiple disparate groups that may not/cannot be anticipated. Furthermore, as both aligned and unaligned peaks are incorporated into the final consensus, we minimize the amount of data lost in this process while enhancing the signal within alignable regions.

This algorithm uses peaks rather than full resolution spectra as the basis for alignment. We consider this to be a general advantage as the datasets are much smaller while having no loss of real spectral information, as peak location, height and width are all maintained. A primary goal of the PCANS process is to provide better input data for multivariate statistical analysis that will help identify significant groups in the sample set. As shown in the OPLS loadings coefficients plots in figure 7, the peak profiles provide an ample representation of the NMR spectra so that specific compounds can be identified. It could be argued that these peak profiles are even easier to interpret than real spectra as they are free from spectral noise and have perfectly uniform linewidths. But, should a more traditional representation of NMR spectra be desired, the information has been maintained to regenerate such a spectrum. In general, the use of peak profiles as input to PCANS allows us to maximize the amount of high-quality information available for alignment and further downstream analysis. We are further investigating the use of more sophisticated peak picking algorithms that will include recognition of peak multiplets and advanced recognition of shoulder peaks that are often present in samples such as serum that displays large, broad peaks due to the presence of macromolecules. While we have used a robust peak picking algorithm, improvements in this step will help minimize issues of inconsistent peak selections across samples. Continued incorporation of more sophisticated scoring schemes as well as more realistic handling of multiplets (rather than as separate peaks), is expected to further enhance the effectiveness of our approach. Finally, while PCANS was developed specifically with NMR data in mind, it has the potential to be applicable to the alignment of other types of data with similar properties. In particular, chromatographic data which is composed of peak positioned along a time axis would be amenable to PCANS alignment. Future work will attempt to further extend these capabilities.

## Methods

### Experimental NMR data collection and processing

Complete details on the urine collection and sample preparation are given in [12]. Briefly, the samples consisted of 540 ml of urine plus 60 ml of a D_2_O solution containing 5 mM trimethylsilylpropionate-d4 (TSP) as a concentration and chemical shift reference. The solutions were transferred to 5 mm NMR tubes and NMR spectra were acquired on a Varian Inova 400 MHz spectrometer using a 5 mm pulsed field gradient, inverse detection probe (Varian, Inc., Palo Alto, CA). The spectra were acquired with 1024 transients and a sweep width of 4650 Hz digitized with 16384 points. The pulse sequence included a 4 second solvent presaturation period and a 2.6 second acquisition time. A 45 degree excitation pulse was used to provide quantitative results.

The data were processed using ACD software version 9 (Advanced Chemistry Development, Toronto, Canada). A 0.1 Hz exponential line broadening was applied to the data. The spectra were phased and baseline corrected using a 6th order polynomial fitting algorithm implemented in the software. The spectra were normalized to the integral for the TSP peak. The digitized spectra were exported as text files for subsequent peak picking prior to alignment with the PCANS method. Spectral binning was carried out by dividing the spectrum into uniform 0.04 ppm bin windows and taking the integral value as the sum of the intensities of all peaks in that bin. The regions from 0.5 to 4.7 ppm and 4.9 to 9.5 were included in the integration. The regions below 0.5 and above 9.5 contained only noise and the region from 4.7 to 4.9 contained the residual solvent peak.

### Multivariate statistical analysis

The statistical analyses were performed using SimcaP+ version 11.5 (Umetrics, Umea, Sweden). Pareto scaling was applied to both the peak picked and binned NMR data prior to principal component analysis (PCA) and orthogonal projection to latent structures - discriminant analysis (OPLS-DA) [13,14].

### Peak picking

The simulated spectra were based upon peaks manually chosen from an actual urine spectrum. Peaks were chosen such that they contained a range of features typically found within normal spectra including tall and small peaks, clusters of many closely spaced peaks, doublets, etc. These peaks and their associated chemical shift position, height and width were then used as the basic material from which simulated spectra (peak profiles) were generated. Thus, the peaks used for simulation are a representative subset of the original spectrum and the simulation program uses sets of these along with defined amounts and types of variation to generate the simulated profiles. For simulated spectra, the steps responsible for peak detection and peak attribute assignment are skipped since the simulated spectra are already defined with a set of peaks with associated attributes.

For real NMR spectra, the peak detection algorithm uses the derivative of the spectrum to detect and define potential peaks. Potential peaks must have a zero first derivative, a negative second derivative and be composed of at least 8 points, where points here refer to data values from the digitized raw spectra. In addition, we use the number of points that define a peak as well as peak height relative to neighbors to determine 'real' peaks from noise peaks within a given spectrum. Specifically, for each potential peak, we look in a region centered around this point (151 points in this work) and call this a 'true' peak if it exceeds a user defined height. This threshold height is based on the height of surrounding points within this region. In this work a true peak had to have a height greater than 70% of the surrounding points. The resulting peaks that define each spectrum are characterized by the attributes of relative intensity (height) at peak apex, chemical shift position at peak apex, and the width at half-height of the peak. The width at half-height is calculated by fitting a triangle to the peak based on the points prior and immediately after the apex point. The base of the triangle estimates the width at half height for the peak. Note that if peaks are not perfect Lorentzians, then relative intensity and width at half-height will not be redundant information.

### The PCANS algorithm

The overall flow of the PCANS alignment algorithm is diagrammed in Figure 1. Detailed algorithm pseudocode for both naive and dynamic programming alignment is provided in Additional File 2. After peak detection, the remaining alignment steps are the same for both real and simulated spectra. The process begins with highly similar pairs of spectra being identified using synchronous sample-sample correlation (Figure 1A) [15]. We note that the statistical correlation between spectra will be more influenced by the larger peaks, but this is simply a starting point in choosing which spectra to try and align first into a consensus spectrum; all peaks will ultimately be aligned. Once the alignment pairs have been identified, the pairwise alignment process begins with naive peak alignment as illustrated in Figure 1B. The naive peak alignment algorithm aligns corresponding peaks within the pair that have ninety percent or greater similarity across all peak attributes. Doing so also generates unaligned regions that are often bounded on both sides by regions composed solely of these highly similar (and easily alignable) peaks.

In both the naive as well as dynamic programing alignment, described next, crossover of peaks is prevented. Here, crossover is defined as shifting a peak over an adjacent peak that has already been aligned to a peak in the paired peak profile. In addition, peaks are restricted by the amount of chemical shift position movement that is allowed based upon a user defined maximum. Therefore, a pair of peaks will only align together if the amount of movement that the peaks need to make for the alignment is less than this user defined maximum. Typically, the user would define this maximum chemical shift position movement as ± 0.04 or ± 0.03 ppm, but the value is data dependent. We note that by aligning each peak within its own user defined window the notion of linear or non-linear shifting of peaks across the spectrum need not be considered.

The next step in alignment involves defining corresponding unaligned segments of the peak profile pair as depicted in panel C of Figure 1. Here, each spectra is segmented such that only the unaligned peaks contained within a segment will be subject to the dynamic programming alignment process. Again, these segments are paired between the two peak profiles and segments are bounded on each side either by already aligned regions or "empty" regions where it is impossible to form an alignment between a pair of peaks based upon the user-defined maximum chemical shift variation.

Both the naive and dynamic programming alignment schemes rely upon a scoring function that determines the similarity between the two corresponding peaks (Equation 1). Note that this similarity score is different from correlation, despite it ranging from 0.0 to 1.0. This score indicates the proportion of similarity between two peaks, i.e. a score of 1.0 indicates the corresponding peaks are exactly the same. The similarity is determined based on the three peak attributes of height at apex, *h*, width at half height, *w*, and chemical shift position, *c*. While in this work each of the three peak attributes are assigned so as to contribute an equal proportion to the score, the assignment of these proportions, *p*_*h*_, *p*_*w *_and *p*_*c*_can readily be altered as appropriate. For both height and width, the similarity is measured by difference of the two values scaled by the larger of the two subtracted from one. For the variation in chemical shift, the similarity is measure by the difference scaled by the user defined maximum amount of acceptable variation between peaks, *m*, subtracted from one.(1)

A modified dynamic programming algorithm is used to align peaks within each of the segments (see Additional File 2 for pseudocode). The algorithm involves using the typical dynamic programming recursion, where the scores assigned for a given alignment between peaks are defined using the scoring function enumerated above with a gap penalty, *gp*, is imposed for unaligned peaks. The modification involves assigning a large penalty, the boundary penalty *bp*, when alignment between two peaks involves chemical shift variation greater than *m *or when two aligned peaks do not achieve the minimum acceptable similarity for alignment, *minScr*. The user defines both of these values, -0.10 *gp *and -5.0 *bp *in this work, with the assignment of the large penalty preventing the algorithm from violating either the maximum allowable chemical shift variation, *m*, or the minimum allowable similarity for alignment.

The recursive formula for aligning a pair of peak profiles with our modified dynamic programming scheme is the following (Equation 2). Given a pair of spectra *S *and *T*, one defines a scores matrix *c *such that *c *has *i *rows equal to length(*S*) + 1 and *j *columns equal to length(*T*) + 1. The function *Scr*(*x, y*) returns the similarity score between two peaks, x and y, computed using the formula above. The gap penalty, *gp*, should be greater in value than the boundary penalty, *bp*. The gap penalty can range from the user defined minimum similarity, *minScr *(0.60 in this work), to a small negative number, typically -1.0. The boundary penalty should be a large negative number (we used -999 in our implementation to allow the algorithm to automatically assign its value).(2)

Panel E of Figure 1 illustrates the final step in the process where the consensus peak profile is formed. Specifically, the consensus profile is generated by assigning a new consensus peak to each successfully aligned pair of peaks, where this consensus peak takes on the median chemical shift value and the average relative height and width of the paired aligned peaks. Peaks from either profile that fail to align are allowed to "pass-through" to the consensus profile and maintain their original attributes. Panel E of Figure 1 depicts successfully aligned peaks as those contained within a shaded box, those that failed alignment are unadorned.

Figure 2 illustrates how the entire process diagrammed in Figure 1 is repeated on the resulting consensus profiles until a single consensus profile is produced. The peak profiles in the top row of figure 2 demonstrate the initial step where the pairs of input profiles are aligned together to form consensus profiles. The next steps involve pairing these resulting consensus profiles together, aligning them and forming 'new' consensus profiles. This process is repeated until only a single consensus profile exists as depicted at the bottom of Figure 2. This final consensus profile is used to adjust the chemical shift positions of the peaks from the original input profiles to their final aligned positions.

For the determination of optimal alignment parameters (*p*_*h*_, *p*_*w *_and *p*_*c *_in the scoring function), we perturbed the peak attributes of the simulated peak profiles in a variety of different ways. Additional File 1: Figure S2 depicts a representative result of one of the many simulations that were run. The results from these experiments indicate that using equal proportions is robust regardless of the perturbations introduced, as long as all three attributes experienced some amount of perturbation. If the amount of perturbation experienced by one of the three attributes is expected to be considerably less than the other two, the user might consider increasing its contribution to the score function.

### Algorithm speed

In our Python implementation, alignment of the described 22 real mouse urine spectra takes approximately 2 minutes on a 2 GHz laptop. Approximately 30 seconds involves the actual process of alignment, with the remaining time involving peak picking and other data processing. Alignment of 150 real mouse urine spectra takes approximately 38 minutes with 1 min 54 sec being involved in alignment.

### Simulation of NMR spectra peak profiles

To generate NMR profiles that were as realistic as possible our simulated peak profiles are based upon characteristics of urine spectra from mice. Specifically, we follow the distribution of peak locations, heights and widths as estimated from murine urine spectra using the spectrum visualization utility implemented in ACD 1D NMR Processor, version 11 (Advanced Chemistry Development, Toronto, Canada). The spectral peaks used for calculating these distributions range in chemical shift position from 2.0 ppm to 4.10 ppm. These distributions are coded into a software utility that allows the generation of simulated peak profiles. In addition, user-defined levels of noise in chemical shift position, height and width can be defined. To help simulate attributes observed with real NMR spectra, the number of peaks generated per spectrum is varied through the addition of noise peaks to the simulated profiles. To evaluate algorithm performance with profiles originating from multiple distinct classes, we generate spectra from distinctly different templates where the user defines the number of peaks common between templates.

## Availability of the software

An implementation of our algorithm, coded in Python, is available at http://gomezlab.bme.unc.edu/tools and as Additional File 3.

## Authors' contributions

JMS, TMO and SMG conceived the project, JMS performed the research, JMS, TMO and SMG analyzed the results. All authors read and approved the final manuscript.

## Supplementary Material

Additional file 1 **Supplemental Figures. Figure S1**. Standard deviations corresponding to the alignment accuracies shown in Figure 3. **Figure S2**. Accuracy of alignment as a function of scoring weights assigned to peak attributes (chemical shift, height, width). **Figure S3**. Loadings plots of simulated peak profiles corresponding to PCA analysis in Figure 5. **Figure S4**. Loadings plots of mouse urine profiles corresponding to PCA analysis in Figure 6. **Figure S5**. Example region (with identified peaks) of mouse urine spectrum used to generate simulated peak profiles. Click here for file

Additional file 2 **Alignment Pseudocode**. Contains description and pseudocode for naive and dynamic programming alignment schemes. Click here for file

Additional file 3 **Source Code**. Contains source code and sample data files. Click here for file
