# Supplementary material for: Enhancing metabolomic data analysis with Progressive Consensus Alignment of NMR Spectra (PCANS)
Source: BMC Bioinformatics. 2010 Mar 9;11:123. doi: 10.1186/1471-2105-11-123 (PMC2851603; doi:10.1186/1471-2105-11-123)
Supplement: Additional file 1 — Supplemental Figures. Figure S1. Standard deviations corresponding to the alignment accuracies shown in Figure 3. Figure S2. Accuracy of alignment as a function of scoring weights assigned to peak attributes (chemical shift, height, width). Figure S3. Loadings plots of simulated peak profiles corresponding to PCA analysis in Figure 5. Figure S4. Loadings plots of mouse urine profiles corresponding to PCA analysis in Figure 6. Figure S5. Example region (with identified peaks) of mouse urine spectrum used to generate simulated peak profiles. [file 1471-2105-11-123-S1.PDF]

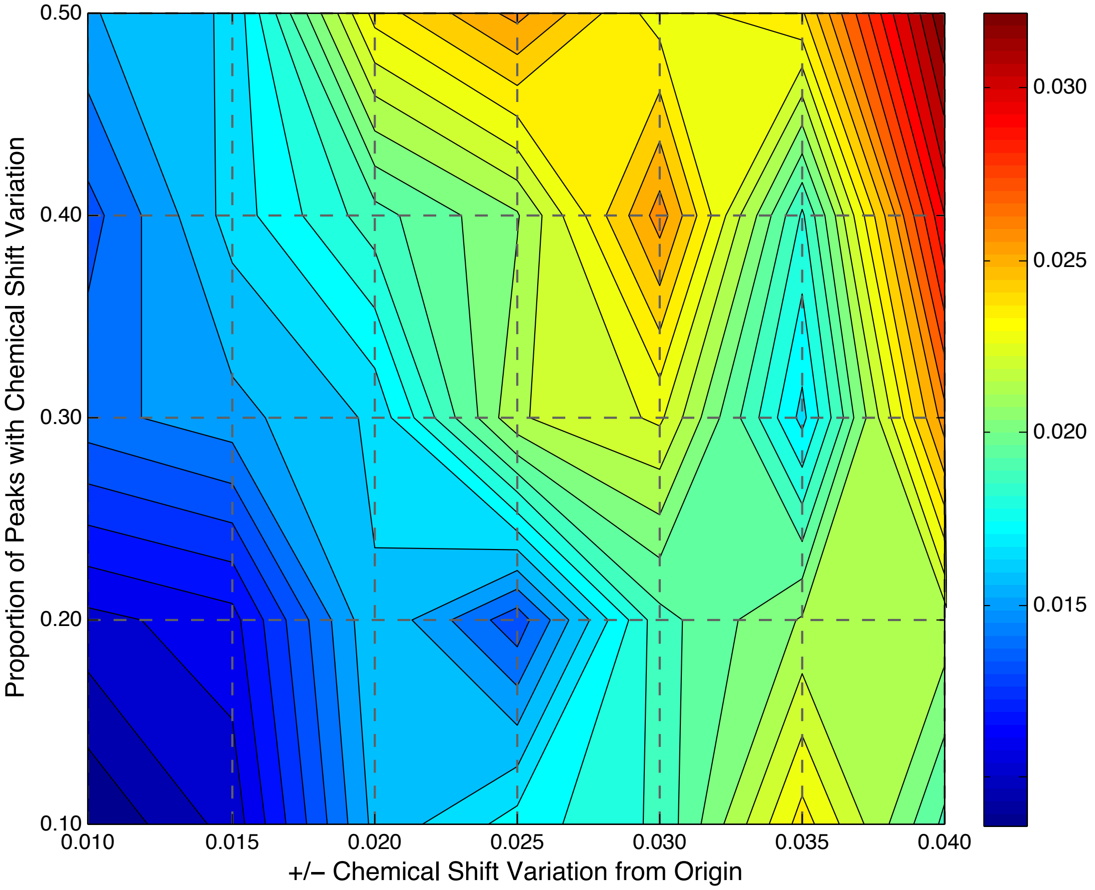

Figure 1: Standard deviations corresponding to the alignment accuracies shown in Figure 2 of the paper. The x-axis indicates ± range of chemical shift variation and the y-axis indicates proportion of peaks per peak profile that experienced chemical shift variation. The graph depicts the standard deviation of the accuracy as indicated by the colorbar on the right. Besides chemical shift position, both relative intensity and width were randomly perturbed by ±10% of the origin for 25% of the peaks within each peak profile and 50% of the profiles had 1-4 noise peaks randomly added.

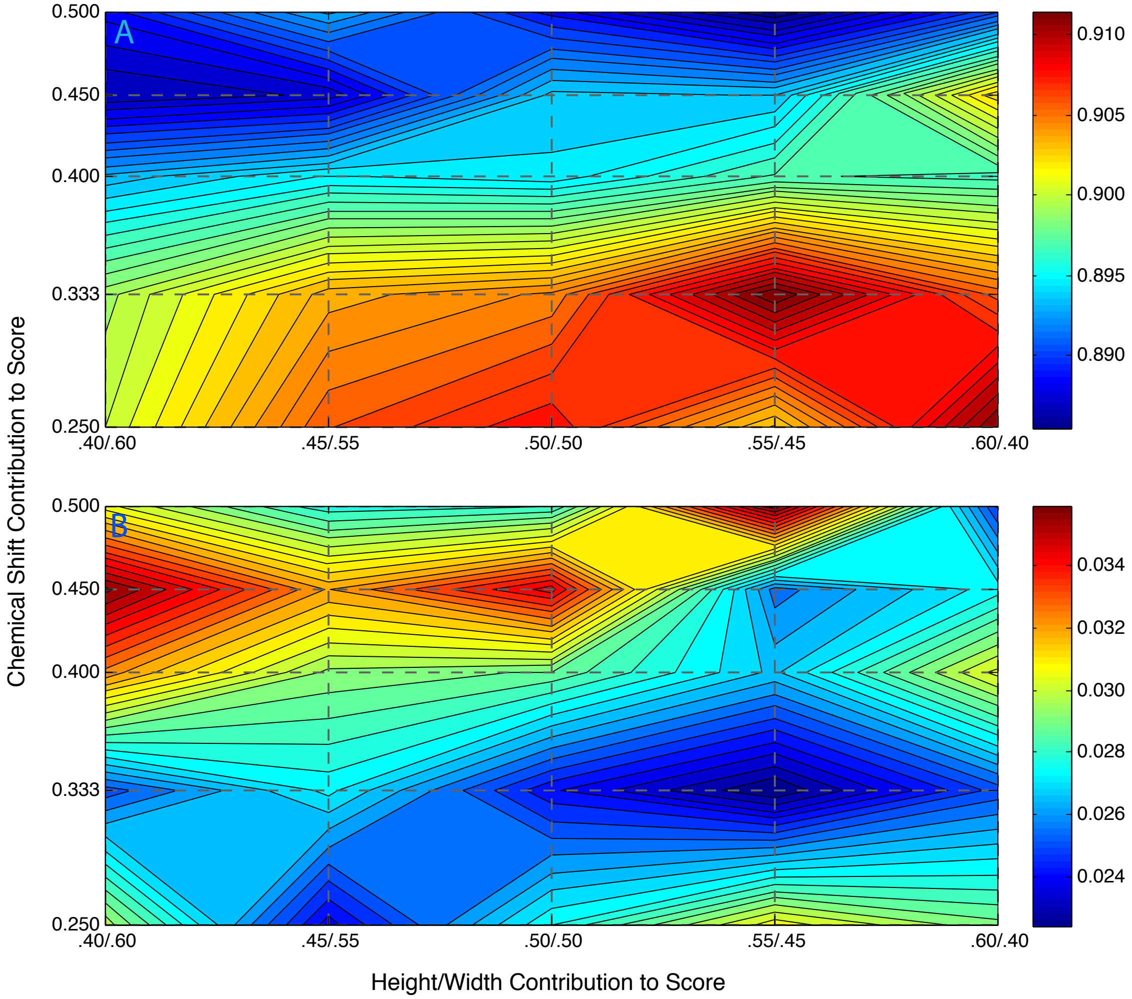

Figure 2: Accuracy of alignment as a function of scoring weights assigned to peak attributes (chemical shift, height, width). Simulated peak profiles were used with ±0.03 variation in chemical shift for 50% of peaks, ±0.10 perturbation in height for 25% of peaks, ±0.10 perturbation in width for 25% of peaks, and 1-4 noise peaks randomly added to 50% of the profiles. The y-axis indicates the proportion of the score that is attributed to chemical shift position, the x-axis indicates the proportion of the height and width that contribute to the remaining proportion of the score. Panel A depicts accuracy as indicated by the colorbar on the right and Panel B depicts the standard deviation of the accuracy measurements shown in panel A.

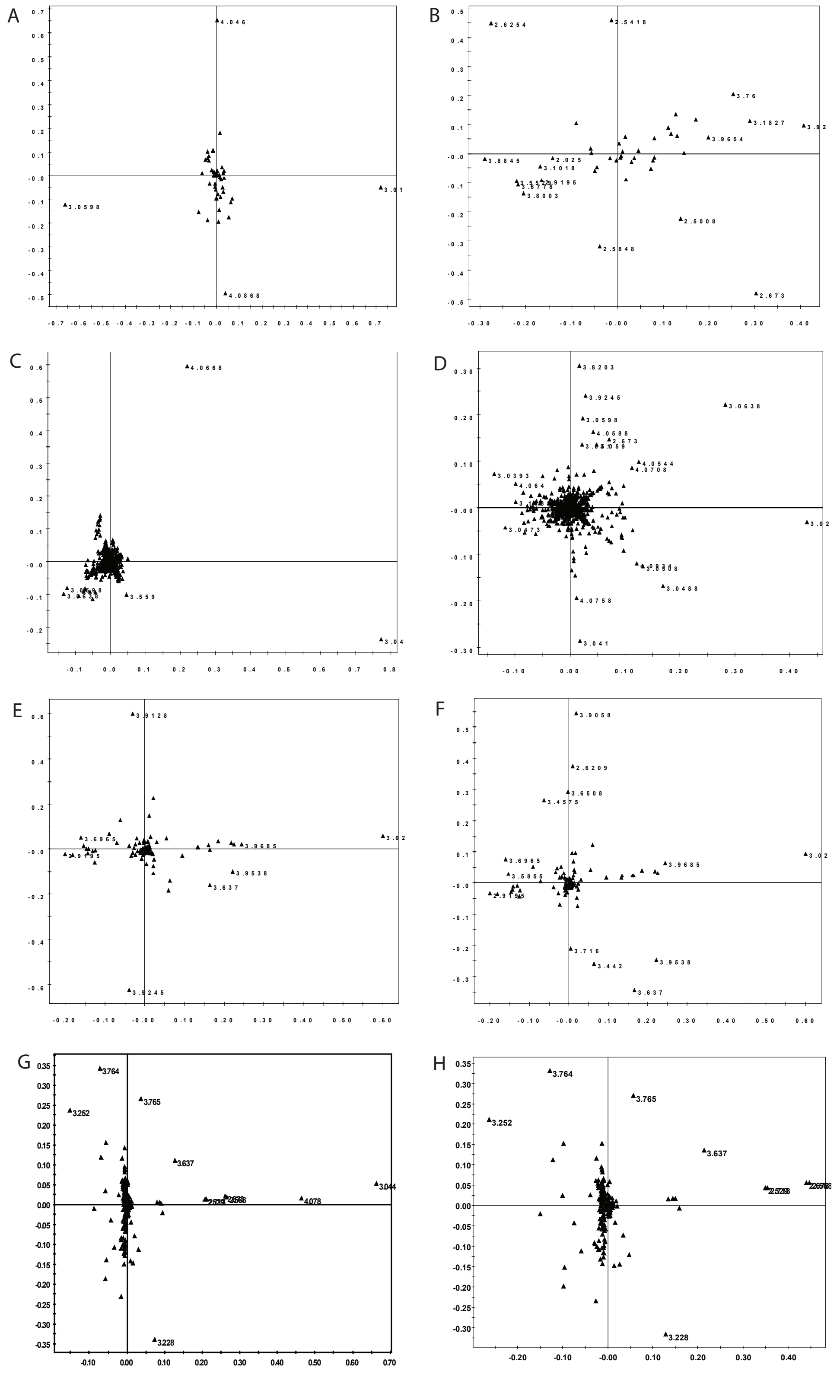

Figure 3: Loadings plots of binned (A & B), unaligned (C & D), PCANS aligned (E & F), and template aligned (G & H) simulated peak profiles, with (B, D, F & H) and without (A, C, E & G) outliers removed.

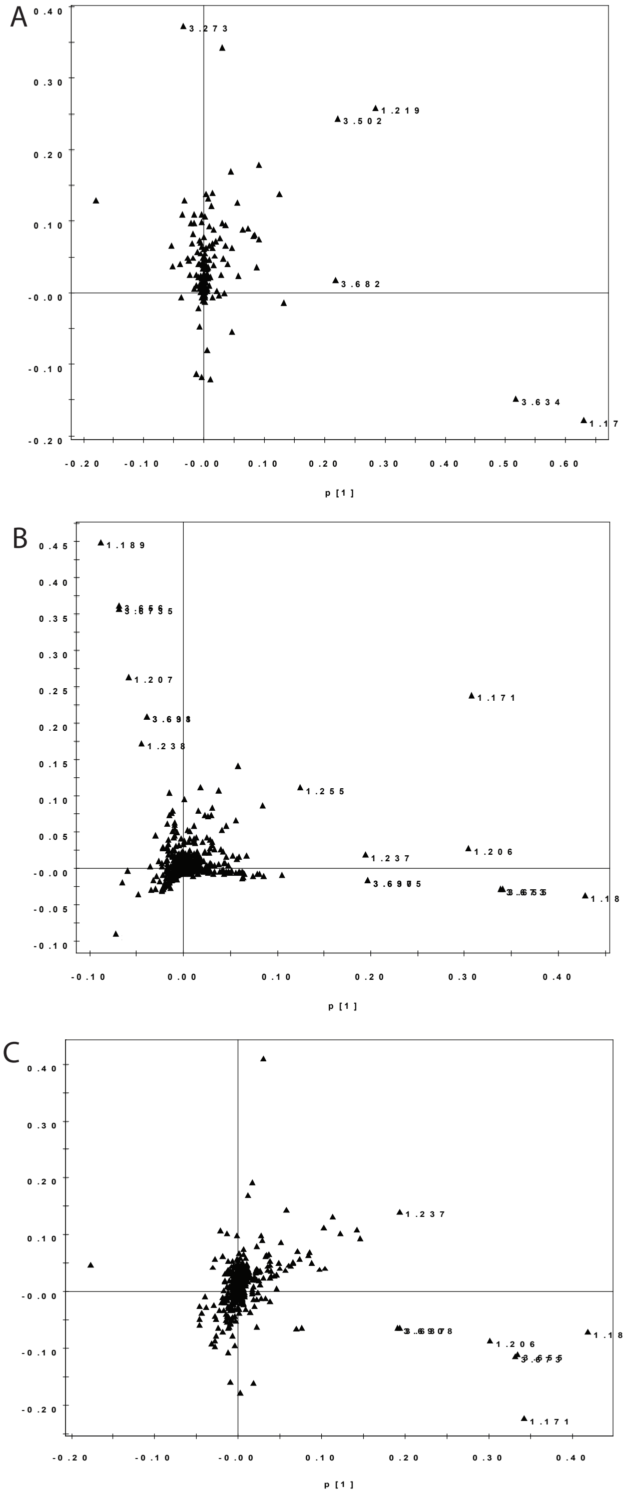

Figure 4: Loadings plots of binned (A), unaligned (B), and aligned (C) mouse urine peak profiles. Peaks associated with EtOH and EtOH-glucuronide are labeled with their chemical shift position.

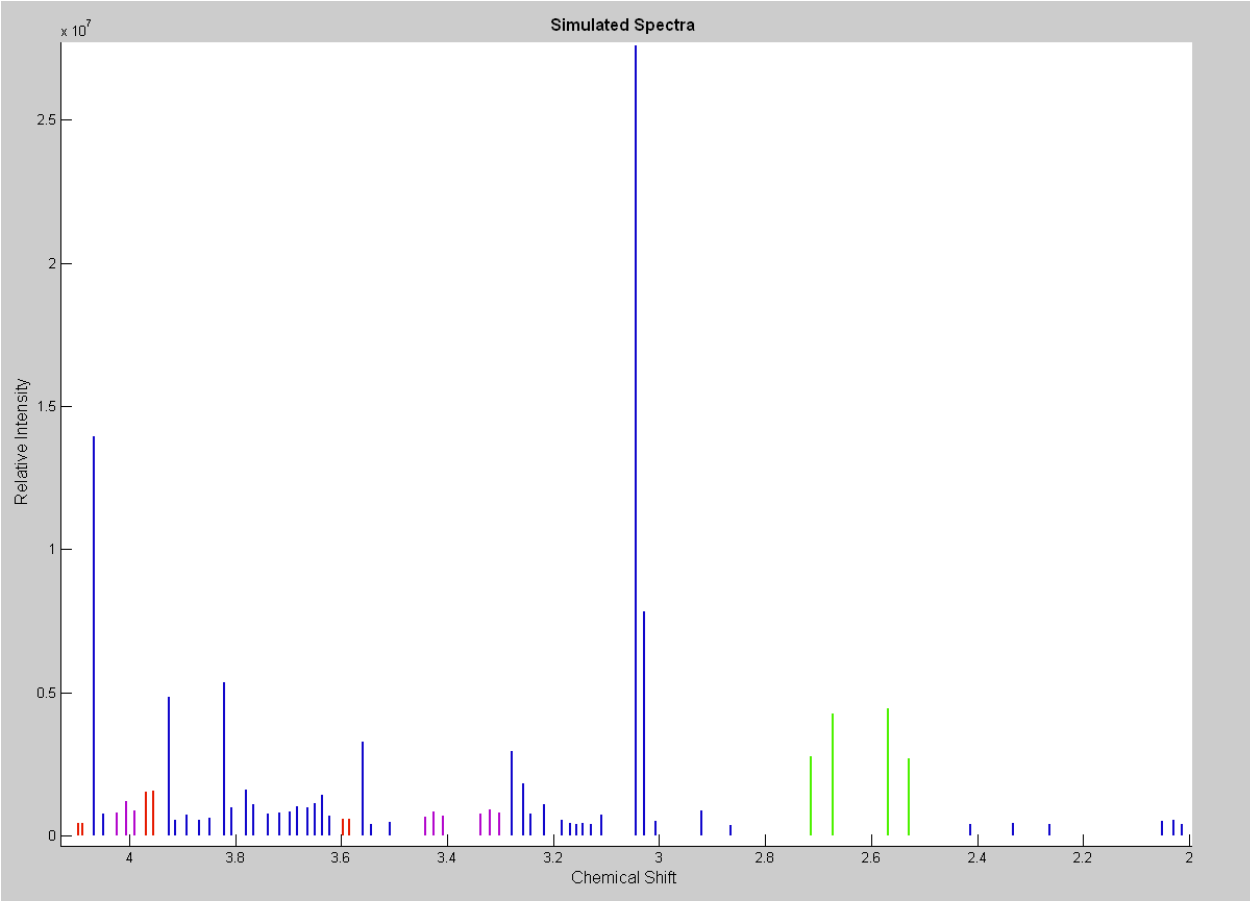

Figure 5: Region (with identified peaks) of mouse urine spectrum used to generate the peaks of the simulated peak profiles. The x-axis indicates chemical shift in ppm and the y-axis indicates relative intensity (height). Peak membership to a spin-spin coupling group is indicated by the peak color; such that singlets are blue, doublets are red, triplets are purple, and quartets are green.
