# Supplementary material for: Enhancing metabolomic data analysis with Progressive Consensus Alignment of NMR Spectra (PCANS)
Source: BMC Bioinformatics. 2010 Mar 9;11:123. doi: 10.1186/1471-2105-11-123 (PMC2851603; doi:10.1186/1471-2105-11-123)
Supplement: Additional file 2 — Alignment Pseudocode. Contains description and pseudocode for naive and dynamic programming alignment schemes. [file 1471-2105-11-123-S2.PDF]

Algorithms

For the alignment algorithms defined below, let A define the set of input peak profiles, where profile X can be defined by peaks y where $\{y \mid A[X, y], 1 \leq y \leq n_X\}$ and the value of n_X is the number of peaks that were picked for peak profile X. The inputs for the pseudocode below involve only aligning a pair of peak profiles, S and T, such that $S, T \in A$, $\{i \mid A[S, i], 1 \leq i \leq n_S\}$ and $\{j \mid A[T, j], 1 \leq j \leq n_T\}$.

Naive Alignment Scheme

The naive alignment incorporates a greedy algorithm that will align two nearby peaks as long as they are close in proximity (chemical shift position) to each other and achieve a high similarity score (i.e. also have high similarity in height and width). The procedure $NaiveAlign(S,T,maxCS,minScoreN)$, naively aligns the pair of peak profiles S and T. This procedure inputs $maxCS$, the chemical shift value that is the maximum the user expects to have to shift a peak to obtain a match, and $minScoreN$, the minimum value of the similarity between two peaks to allow for naive alignment. Additionally, the value of $minScoreN$ is used to define the required amount of similarity in chemical shift position that two peaks must have to allow naive alignment.

The similarity between two peaks is calculated using the function $CalcScore(S[i], T[j], maxCS)$ which is based on the similarity score formula presented in the methods section of the paper. Typically, $minScoreN$ should be a high value of 0.88 or greater (0.90 for this paper) and $maxCS$ should range within 0.04 - 0.02 ppm (0.04 for this paper). Naive alignments are made using the procedure $MakeNaiveMatch(S,T,sIdx,tIdx)$, which is not illustrated below due to its reliance on our algorithm's spectra data structure. The $MakeNaiveMatch$ procedure makes the naive matches given the input pair of peak profiles and the indices of their peaks that match. Nothing is returned, but the underlying peak profile data structure is changed to reflect the naive matches. Pseudocode for the algorithm can be found below as Algorithm 1 for naive alignment with the two helper functions defined in Algorithm 2.

Dynamic Programming Alignment Scheme

To dynamically align the pair of peak profiles S and T, the procedure $DynProgAlign(S,T,maxCS,gp,bp,$ $minScoreD)$ uses the recursive formula defined in the methods section of the paper. The recursive formula from the paper defines an alignment scores matrix $c[i, j]$ and the backtrack matrix $b[i, j]$ that indicate the optimal solution. Notice that indices i and j from these matrices (scores and backtrack) are defined as i=0,...,n_S and j=0,...,n_T. The pseudocode in Algorithm 3 follows a modified dynamic programming

alignment scheme as outlined by the recursive formula in the paper.

The function $CalcScore(S[i], T[j], maxCS, minScoreD)$ calculates the similarity score between two peaks using the similarity score formula as indicated in the methods section of the paper. A gap penalty, gp, is incurred each time two peaks fail to align. A boundary penalty, bp, is incurred each time two peaks are so dissimilar that they should not be allowed to align together based on the user defined minimum similarity required for dynamic alignment, $minScoreD$, or if two peaks' difference in chemical shift position is greater than the maximum allowable chemical shift variation, $maxCS$. The gap penalty, gp, and boundary penalty, bp, can be input by the user.

Typically, $minScoreD$ is a high value of 0.50 or greater, but this parameter should be set based upon the user's discretion (for this paper 0.60). The gap penalty (-0.10 for this paper) should be greater in value than the boundary penalty (-5.0 for this paper). The gap penalty can range from the user defined minimum similarity, $minScoreD$, to a small negative number, typically -1.0. The boundary penalty should be a large negative number or set to -999 to allow the algorithm to automatically set the value.

Pseudocode for the algorithm can be found below as Algorithm 3. Notice for simplicity the $CalcScore(S[i], T[j], maxCS, minScoreD)$ function in the algorithm is implemented in a manner that returns a similarity score that is less than $minScoreD$ if the difference in chemical shift position is greater than the maximum allowable shift, $maxCS$. This allows the boundary penalty to be incurred when it is appropriate.

Algorithm 1

NaiveAlign$(S, T, maxCS, minScoreN) \equiv$
$m \leftarrow length[S]$
$ssT \leftarrow 1$
$searchCS \leftarrow maxCS * (1.0 - minScoreN)$
<u>for</u> $i \leftarrow 1$ **<u>to</u>** m **<u>do</u>**
    $temp \leftarrow ReturnStart(S, T, searchCS, i, ssT)$
    <u>if</u> $temp \neq -999$
      <u>then</u> $ssT \leftarrow temp$
          $matchI \leftarrow ReturnMaxMatchIdx(S, T, maxCS, searchCS, i, ssT, minScoreN)$
          <u>if</u> $matchI \neq -999$
            <u>then</u> $MakeNaiveMatch(S, T, i, matchI)$
          <u>fi</u>
    <u>fi</u>
<u>end</u>

Algorithm 2

```
ReturnStart(S, T, searchCS, idxS, ssT) ≡
n ← length[T]
z ← ssT
if T[z].chemShift ≥ (S[idxS].chemShift − searchCS)
  then
        while (z > 1 and T[z].chemShift > (S[idxS].chemShift − searchCS)) do
              z ← z − 1
        end
        if (z ≥ 1 and T[z].chemShift ≤ (S[idxS].chemShift − searchCS))
          then if T[z].chemShift = (S[idxS].chemShift − searchCS)
                 then rssT ← z
                  else rssT ← z + 1
               fi
           else rssT ← −999
        fi
   else
        while (z < n and T[z].chemShift < (S[idxS].chemShift + searchCS)) do
              z ← z + 1
        end
        if (z ≤ n and T[z].chemShift ≥ (S[idxS].chemShift + searchCS))
          then if T[z].chemShift = (S[idxS].chemShift + searchCS)
                 then rssT ← z
                  else rssT ← z − 1
               fi
           else rssT ← −999
        fi
fi
return rssT
ReturnMaxMatchIdx(S, T, maxCS, searchCS, idxS, ssT, minScoreN) ≡
n ← length[T]
z ← ssT
bestV ← CalcScore(S[idxS], T[z])
bestI ← z
z ← z + 1
while (z ≤ n and T[z].chemShift ≤ (S[idxS].chemShift + searchCS)) do
      if bestV < CalcScore(S[idxS], T[z], maxCS)
        then bestV ← CalcScore(S[idxS], T[z], maxCS)
             bestI ← z
      fi
      z ← z + 1
end
if bestV < minScoreN
  then bestI ← −999
fi
return bestI
```

Algorithm 3

```
DynProgAlign(S, T, maxCS, gp, bp, minScoreD) ≡
m ← length[S]
n ← length[T]
if bp == −999
  then bp = gp ∗ n ∗ m
fi
for i ← 0 to m do
    for j ← 0 to n do
        if i = 0 or j = 0
          then c[i, j] ← i ∗ gp + j ∗ gp
                if i = 0
                  then b[i, j] ← " ← "
                   else b[i, j] ← " ↑ "
                fi
          else DiagScore ← CalcScore(S[i], T[j], maxCS, minScoreD)
                if DiagScore < minScoreD
                  then DiagScore ← bp
                fi
                if (c[i − 1, j − 1] + DiagScore ≥ c[i − 1, j] + gp) and
                   (c[i − 1, j − 1] + DiagScore ≥ c[i, j − 1] + gp)
                  then c[i, j] ← c[i − 1, j − 1] + DiagScore
                       b[i, j] ← " ↖ "
                       if  else (c[i − 1, j] + gp ≥ c[i − 1, j − 1] + DiagScore) and
                                (c[i − 1, j] + gp ≥ c[i, j − 1] + gp)
                           then c[i, j] ← c[i − 1, j] + gp
                                b[i, j] ← " ↑ "
                            else c[i, j] ← c[i, j − 1] + gp
                                b[i, j] ← " ← "
                       fi
                fi
        fi
    end
end
```
