# Supplementary material for: Enhancing metabolomic data analysis with Progressive Consensus Alignment of NMR Spectra (PCANS)
Source: BMC Bioinformatics. 2010 Mar 9;11:123. doi: 10.1186/1471-2105-11-123 (PMC2851603; doi:10.1186/1471-2105-11-123)
Supplement: Additional file 3 — Source Code. Contains source code and sample data files. [file 1471-2105-11-123-S3.ZIP › PCANS/README_PCANS_v2.doc]

**Required Software to Run PCANS GUI:**

1. Python 2.5 (version 2.5.4 is available for download here 🡺 http://www.python.org/download/releases/2.5.4/
2. Python Modules: sys, time, cPickle, Tkinter, string, os, **numpy**, csv, random, math All the modules are included in the standard 2.5 Python package except **numpy** version 1.3.0 can be found here 🡺 <http://sourceforge.net/projects/numpy/files/NumPy/>

* Enthought Python Distribution (EPD Py25 v4.3.0 ) installed for free for academic use includes Python 2.5 and all needed modules

**How to Run PCANS:**

1. Download & unzip PCANS.zip (Windows users ignore the __MACOSX folder that gets created when you unzip PCANS.zip).
2. Load required software (Python 2.5 and then Numpy)
3. Open Terminal window (Command window for Windows users) and move to the …/PCANS/src/ (windows OS: …\PCANS\src\) directory. You are in the correct directory if it contains python program PCANS_AlignmentGUI.py
4. In the terminal window Type “python PCANS_AlignmentGUI.py” to run alignment program – The GUI window below will appear. ***This will not work for Windows OS.*🡺**For a windows OS: type “**C:\Python25\**python PCANS_AlignmentGUI.py” where the “**C:\Python25\**” directory contains the python.exe file. If you have done the standard load of Python 2.5, then python.exe should be located in the “**C:\Python25\**” directory. If you have loaded python.exe somewhere else on your Windows OS you need to replace “**C:\Python25\**” with that location.
5. Default values are contained within the GUI window. The default values will run the alignment on …/PCANS/data/finalMouse_data.csv file (windows OS: …\PCANS\data\ytfinalMouse_data.csv) and output the results to …/PCANS/outputFiles/ directory (windows OS: …\PCANS\outputFiles\). To run an alignment on a different CSV file, one would need to replace “…/PCANS/data/finalMouse_data.csv”(#3) with the location and file name of the different CSV file. In addition, to save the alignment results in a different location one would need to replace “…/PCANS/outputFiles/”(#8) with that new location. The other alignment options can also be changed as described below. Once one has the desired values in the GUI click the ‘OK’ button to run alignment program. Clicking ‘Cancel’ closes the program without running. NOTE THE GUI DOES NO ERROR CHECKING ON INPUTS, MISTAKES WILL LEAD TO A RUNTIME FAILURE. BE AWARE OF WHAT IT ENTERED INTO GUI TEXT BOXES. CLICKING “OK” WITHOUT CHANGING THE DEFAULTS WILL ALWAYS RUN THE PROGRAM.

**Detailed Description of GUI Inputs:**

1. Maximum expected chemical shift. Should be a real number, typically 0.03 or 0.04 ppm depending on the data.
2. Minimum separation expected between peaks within each spectra. Generally 0.001 ppm but is data dependent, could be as small as 0.0001 ppm.
3. Input Spectra File location and file name. Defaults to …/PCANS/data/finalMouse_data.csv OR …\PCANS\data\finalMouse_data.csv, where … represents where PCANS.zip was unzipped. To change to a different input file format should be /MyPath/MySpectraData.csv or C:\MyPath\MySpectraData.csv dependent upon one’s OS. Expects the data to be a csv file, where first column contains chemical shift positions and the rest of the columns contain the spectra. The format should be the same as what can be found in …/PCANS/data/finalMouse_data.csv use it as a guide to creating your input spectra files
4. Number of input spectra in the input spectra file (integer), …/PCANS/data/finalMouse_data.csv has 23 columns first is chemical shift rest are spectra so its input is 22
5. Number of points required to form a peak within a spectra using the peak picking algorithm (integer). Data dependent, but 8 worked well with finalMouse_data.csv.
6. Number of points that define the region immediately surrounding a possible peak (neighbors of a possible peak) within the peak picking algorithm. Select an odd integer of 101 or greater points. 151 worked well with finalMouse_data.csv, which contained a total of 11,901 points from 0.5 to 9.5 ppm
7. Based on relative intensity of the points immediately surrounding a possible peak (neighbors). Peaks that are kept must have relative intensity (height) greater than X proportion of points surrounding it (its neighbors), where X is the proportion entered in this text box. Typically a real number of 0.60 or greater, 0.70 worked well with finalMouse_data.csv.
8. Output directory (including the last backward or forward slash) where output files will be stored. Defaults to …/PCANS/outputFiles/ OR …\PCANS\outputFiles\, where … represents where PCANS.zip was unzipped. To change to a different output directory, the format should be /MyPath/OutputFolder/ or C:\MyPath\OutputFolder\ dependent upon one’s OS.
9. Gap penalty should be a small real number that is less than the minimum expected similarity for a match. Can range from a small negative number to a value that is less than the minimum expected similarity for a match value (#11). -0.10 worked well for finalMouse_data.csv.
10. Boundary Penalty should be a value that is smaller than the gap penalty. If the user would prefer that the algorithm compute this penalty, then set the value to the default of -999. For finalMouse_data.csv this value was set to -999.
11. Minimum expected similarity between two peaks to allow for a match. Should be some proportion that ranges between 0.0 and 1.0. Typical value would be 0.60 or higher, but is data dependent. For finalMouse_data.csv 0.60 was used.
12. Minimum similarity required for naïve alignment, if two peaks are at least this similar then they will be aligned using the naïve alignment scheme. Typically this value would be 0.90 or higher, 0.90 was used for finalMouse_data.csv.
13. Zero fill value is the value that will be used to fill in the output files when there is no peak for a given chemical shift value. Typically we have used 0.0001 instead of zero for the OPLS analysis.

**Expected Output Files in Output Directory:**

1. **origSpectra.csv** – comma separated file that contains input peak profiles (spectra) prior to alignment after peaks have been picked. The first column contains chemicals shift position, next columns contain the relative intensity (height) of the apex of the picked peaks for the input spectra prior to alignment. Zero fill values appear where there are no peaks for a given chemical shift and input profiles (spectra). The final column contains a count of how many peaks exist for a given chemical shift position across all input peak profiles (spectra).
2. **origSpectra.py –** Prior to alignment after peaks have been picked, the input peak profiles (spectra) saved as a spectra object.
3. **alignedSpectraHt.csv --**  comma separated file that contains the input peak profiles (spectra) after PCANS alignment. The first column contains chemicals shift position, next columns contain the relative intensity (height) of the apex of the picked peaks for the input peak profiles (spectra) after alignment. Zero fill values appear where there are no peaks for a given chemical shift and input profiles (spectra). The final column contains a count of how many peaks exist for a given chemical shift position across all input peak profiles (spectra).
4. **alignedSpectraWd.csv --**  comma separated file that contains the input peak profiles (spectra) after PCANS alignment. The first column contains chemicals shift position, next columns contain the width at half-height (width) of the picked peaks for the input spectra after alignment. Zero fill values appear where there are no peaks for a given chemical shift and input peak profiles (spectra). The final column contains a count of how many peaks exist for a given chemical shift position across all input profiles (spectra).
5. **finalConsensusSpectrum.csv --**  comma separated file that contains the final consensus spectrum post alignment. The first column contains chemicals shift position of the peaks in the final consensus spectrum. The values in this column represent the median chemical shift position of all the peaks that were aligned to that chemical shift position using PCANS. The next column contains the average relative intensity (height) of the peaks that were aligned to that chemical shift position in the consensus spectrum, the next column contains the average width at half-height (width) of all the peaks that were aligned to that chemical shift position in the consensus spectrum, and the final column contains the number of peaks that were aligned to that chemical shift position in the consensus spectrum.
6. **finalConsensusSpectrum.py –** Final consensus spectrum post-alignment saved as a spectra object**.**

**Uses UNC’s Copyright and Permission Notice:**

COPYRIGHT AND PERMISSION NOTICE

**UNC Software:** PCANS

Copyright (C) 2009 The University of North Carolina at Chapel Hill
All rights reserved

The University of North Carolina at Chapel Hill (“UNC”) and the developers (“Developers”) of PCANS (“Software”) give recipient (“Recipient”) and Recipient’s Institution (“Institution”) permission to use and copy the software in source and binary forms, with or without modification for **non-commercial purposes only** provided that the following conditions are met:

1) All copies of Software in binary form and/or source code, related documentation and/or other materials provided with the Software must reproduce and retain the above copyright notice, this list of conditions and the following disclaimer.
2) Recipient and Institution shall not distribute Software to any third parties.
3) The Software is provided “As Is.” The Developers can not guarantee the provision of technical support or consultation for the Software. The Developers may provide a location on a UNC Web Site for Recipients to post comments, questions, and suggestions at some time in the future. Recipient may provide the Developers with feedback on the use of the Software in their research at that time. The Developers and UNC are permitted to use any information Recipient provides in making changes to the Software.
4) Recipient acknowledges that the Developers, UNC and its licensees may develop modifications to Software that may be substantially similar to Recipient’s modifications of Software, and that the Developers, UNC and its licensees shall not be constrained in any way by Recipient in UNC’s or its licensees’ use or management of such modifications. Recipient acknowledges the right of the Developers and UNC to prepare and publish modifications to Software that may be substantially similar or functionally equivalent to your modifications and improvements, and if Recipient or Institution obtains patent protection for any modification or improvement to Software, Recipient and Institution agree not to allege or enjoin infringement of their patent by the Developers, UNC or any of UNC’s licensees obtaining modifications or improvements to Software from the UNC or the Developers.
5) Recipient and Developer will acknowledge in their respective publications the contributions made to each other’s research involving or based on the Software. The current citations for Software are:

*<<current citation pending.>>>*

6) Any party desiring a license to use the Software for commercial purposes shall contact The Office of Technology Development at UNC at 919-966-3929.

THIS SOFTWARE IS PROVIDED BY THE COPYRIGHT HOLDERS, CONTRIBUTORS, AND THE UNIVERSITY OF NORTH CAROLINA AT CHAPEL HILL "AS IS" AND ANY EXPRESS OR IMPLIED WARRANTIES, INCLUDING, BUT NOT LIMITED TO, THE IMPLIED WARRANTIES OF MERCHANTABILITY AND FITNESS FOR A PARTICULAR PURPOSE ARE DISCLAIMED. IN NO EVENT SHALL THE COPYRIGHT OWNER, CONTRIBUTORS OR THE UNIVERSITY OF NORTH CAROLINA AT CHAPEL HILL BE LIABLE FOR ANY DIRECT, INDIRECT, INCIDENTAL, SPECIAL, EXEMPLARY, OR CONSEQUENTIAL DAMAGES (INCLUDING, BUT NOT LIMITED TO, PROCUREMENT OF SUBSTITUTE GOODS OR SERVICES; LOSS OF USE, DATA, OR PROFITS; OR BUSINESS INTERRUPTION) HOWEVER CAUSED AND ON ANY THEORY OF LIABILITY, WHETHER IN CONTRACT, STRICT LIABILITY, OR TORT (INCLUDING NEGLIGENCE OR OTHERWISE) ARISING IN ANY WAY OUT OF THE USE OF THIS SOFTWARE, EVEN IF ADVISED OF THE POSSIBILITY OF SUCH DAMAGE.
